# Supplementary material for: The Role of CCL24 in Primary Sclerosing Cholangitis: Bridging Patient Serum Proteomics to Preclinical Data
Source: Cells. 2024 Jan 23;13(3):209. doi: 10.3390/cells13030209 (PMC10854794; doi:10.3390/cells13030209)
Supplement: Supplementary file 1 [file cells-13-00209-s001.zip › Cells_Supp_revised.pdf]

Supplemental Data

Fig. S1

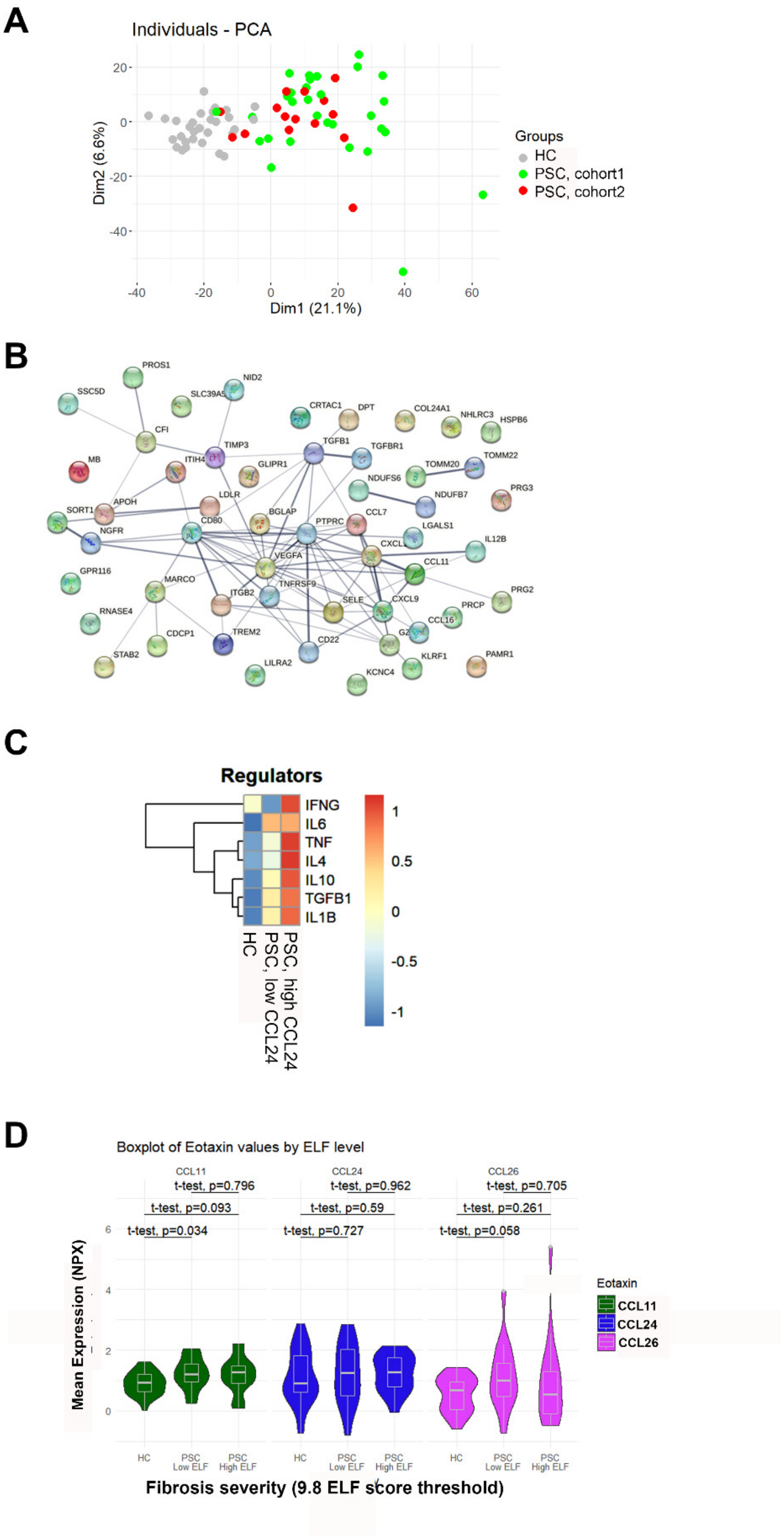
A
Individuals - PCA
Dim2 (6.6%)
Dim1 (21.1%)
20
0
-20
-40
-40
-20
0
20
40
60
Groups
HC
PSC, cohort1
PSC, cohort2
B
PROS1
NID2
SSC5D
SLC39A5
CRTAC1
DPT
COL24A1
NHLRC3
HSPB6
CFI
TIMP3
TGFB1
TGFBR1
TOMM22
MB
ITIH4
GLIPR1
TOMM20
NDUFS6
PRG3
NDUFB7
APOH
LDLR
SORT1
BGLAP
CCL7
CD80
PTPRC
LGALS1
NGFR
IL12B
VEGFA
CCL11
GPR116
MARCO
TNFRSF9
PRG2
ITGB2
SELE
CXCL9
PRCP
RNASE4
CCL16
CDCP1
TREM2
CD22
STAB2
KLRF1
PAMR1
LILRA2
KCNC4
C
Regulators
IFNG
IL6
TNF
IL4
IL10
TGFB1
IL1B
HC
PSC, low CCL24
PSC, high CCL24
1
0.5
0
-0.5
-1
D
Boxplot of Eotaxin values by ELF level
CCL11
t-test, p=0.796
t-test, p=0.093
t-test, p=0.034
CCL24
t-test, p=0.962
t-test, p=0.59
t-test, p=0.727
CCL26
t-test, p=0.705
t-test, p=0.261
t-test, p=0.058
Mean Expression (NPX)
6
4
2
0
HC
PSC Low ELF
PSC High ELF
Eotaxin
CCL11
CCL24
CCL26
Fibrosis severity (9.8 ELF score threshold)

Fig S1. PSC-related canonical pathways are upregulated in patients with PSC and associated with CCL24 levels. (A) Score plots of principal component analysis of proteome profiles in HC cohort and two cohorts of Patients with PSC. (B) Protein-protein interaction network of top 50 proteins that positively correlate with CCL24. (C) Serum levels of the upstream regulators presented as hierarchicaly-clustered heatmap of scaled expression. (D) Expression levels of CCL11, CCL24 and CCL26, stratified by ELF score. Boxes represent interquartile ranges with medians (n = 15-18).

Fig. S2

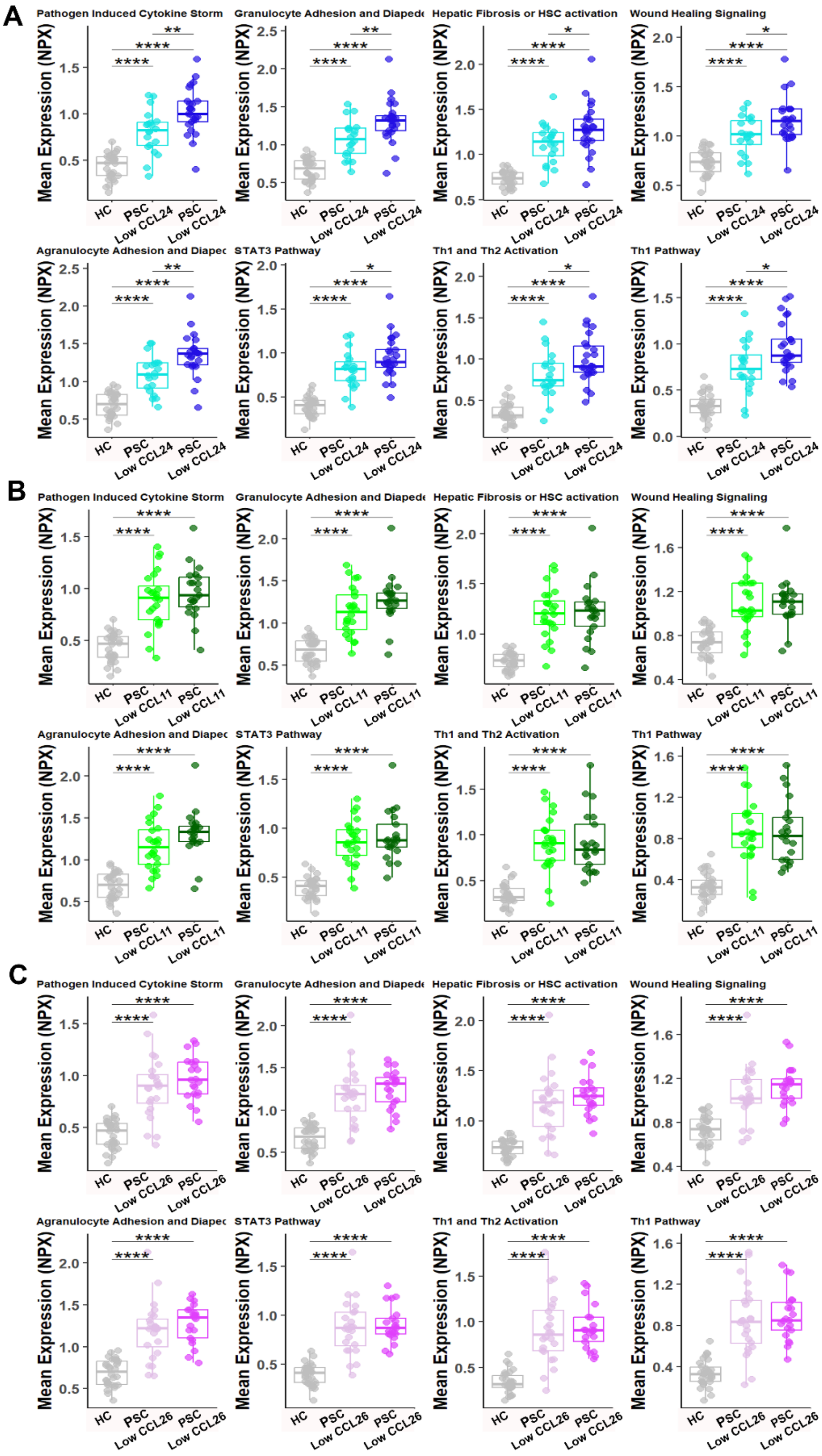

A
Pathogen Induced Cytokine Storm
Granulocyte Adhesion and Diapede
Hepatic Fibrosis or HSC activation
Wound Healing Signaling
Agranulocyte Adhesion and Diapec
STAT3 Pathway
Th1 and Th2 Activation
Th1 Pathway
Mean Expression (NPX)
HC
PSC Low CCL24
PSC Low CCL24
B
Pathogen Induced Cytokine Storm
Granulocyte Adhesion and Diapede
Hepatic Fibrosis or HSC activation
Wound Healing Signaling
Agranulocyte Adhesion and Diapec
STAT3 Pathway
Th1 and Th2 Activation
Th1 Pathway
HC
PSC Low CCL11
PSC Low CCL11
C
Pathogen Induced Cytokine Storm
Granulocyte Adhesion and Diapede
Hepatic Fibrosis or HSC activation
Wound Healing Signaling
Agranulocyte Adhesion and Diapec
STAT3 Pathway
Th1 and Th2 Activation
Th1 Pathway
HC
PSC Low CCL26
PSC Low CCL26

Fig S2. PSC-related canonical pathways are upregulated in patients with PSC and associated with CCL24 levels. The average expression of protein signatures of specific canonical pathways is presented in HC vs. patients with PSC; patients with PSC are stratified by mean expression of each of the three eotaxins: CCL24 (A), CCL11 (B) and CCL26 (C). Boxes represent interquartile ranges with medians ($n = 20\text{-}30$). *, $p < 0.05$; **, $p < 0.01$; ****, $p < 0.0001$. HC, healthy controls.

Fig. S3

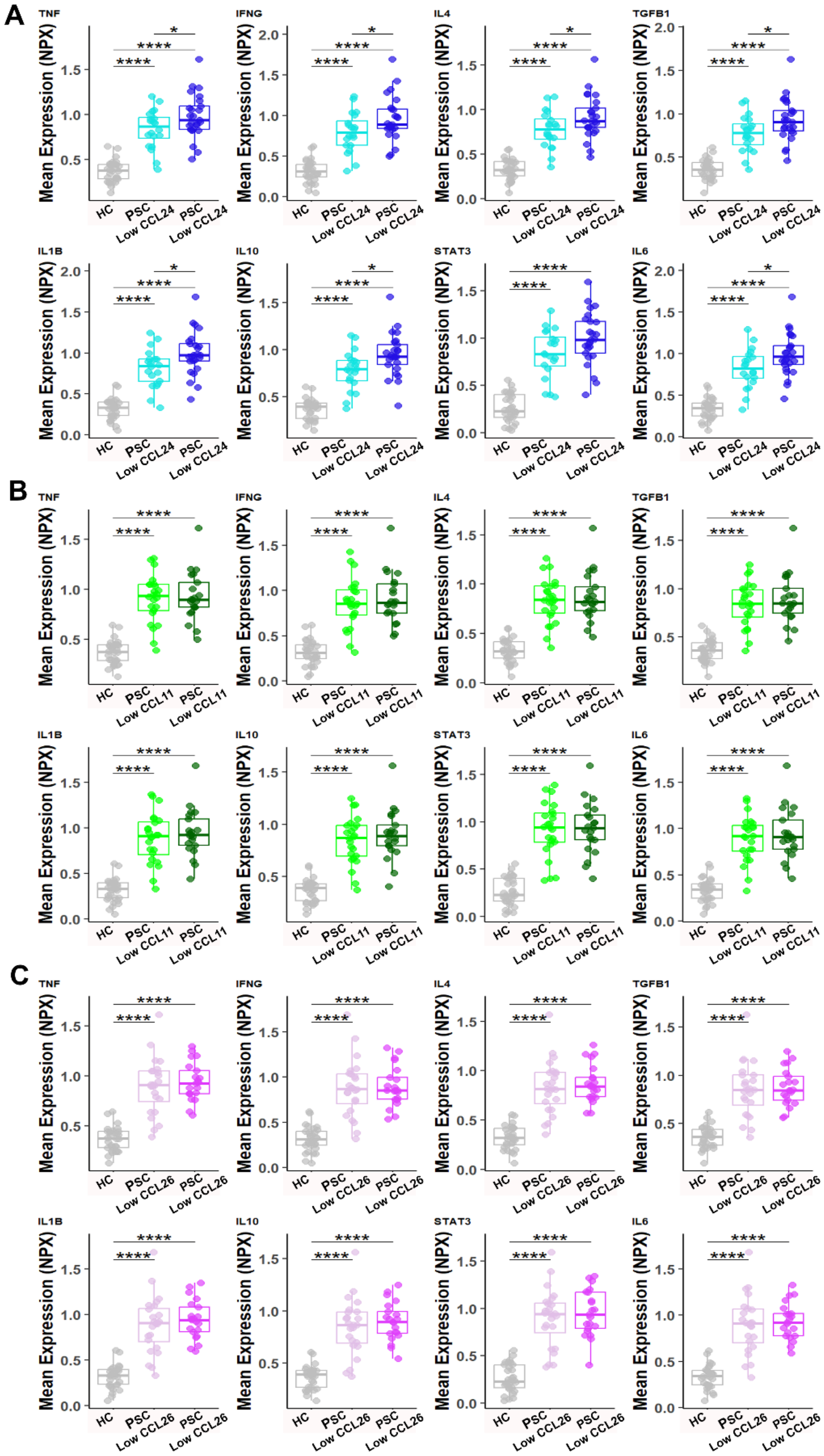
A
TNF
IFNG
IL4
TGFB1
IL1B
IL10
STAT3
IL6
Mean Expression (NPX)
HC
PSC Low CCL24
PSC Low CCL24
B
TNF
IFNG
IL4
TGFB1
IL1B
IL10
STAT3
IL6
Mean Expression (NPX)
HC
PSC Low CCL11
PSC Low CCL11
C
TNF
IFNG
IL4
TGFB1
IL1B
IL10
STAT3
IL6
Mean Expression (NPX)
HC
PSC Low CCL26
PSC Low CCL26

Fig S3. PSC-related upstream regulators are upregulated in patients with PSC and associated with CCL24 levels. The average expression of protein signatures of specific upstream regulators is presented in HC vs. patients with PSC; patients with PSC are stratified by mean expression of each of the three eotaxins: CCL24 (A), CCL11 (B) and CCL26 (C). Boxes represent interquartile ranges with medians (n = 20-30). *, $p < 0.05$; **, $p < 0.01$; ****, $p < 0.0001$.

Fig. S4

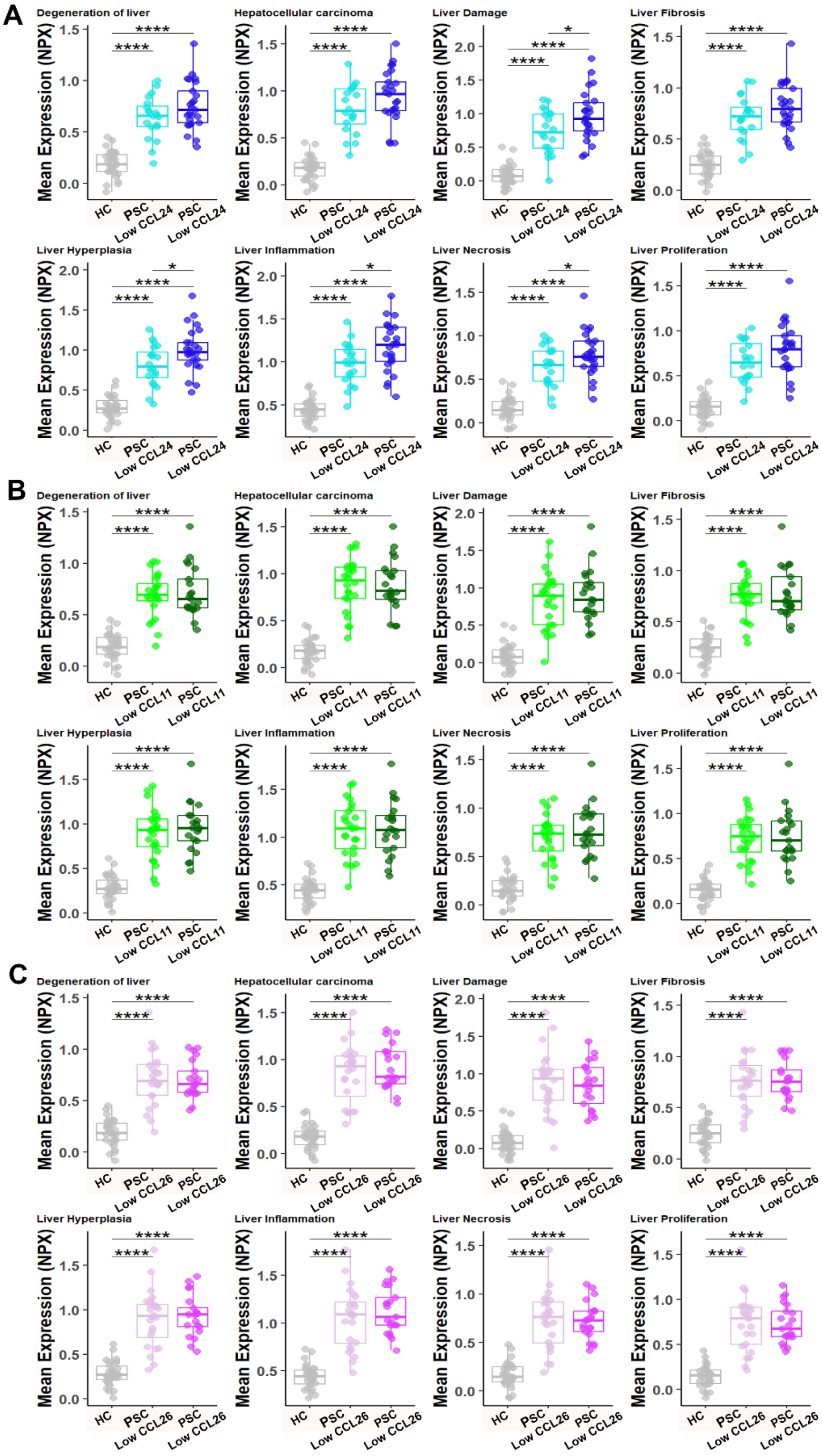
A
Degeneration of liver
Hepatocellular carcinoma
Liver Damage
Liver Fibrosis
Liver Hyperplasia
Liver Inflammation
Liver Necrosis
Liver Proliferation
Mean Expression (NPX)
HC
PSC
Low CCL24
B
Low CCL11
C
Low CCL26

Fig S4. PSC-related toxicity functions are upregulated in patients with PSC and associated with CCL24 levels. The average expression of protein signatures of specific liver-related toxicity functions is presented in HC vs. patients with PSC; patients with PSC are stratified by mean expression of each of the three eotaxins: CCL24 (A), CCL11 (B) and CCL26 (C). Boxes represent interquartile ranges with medians (n = 20-30). *, $p < 0.05$; **, $p < 0.01$; ****, $p < 0.0001$.

Fig. S5

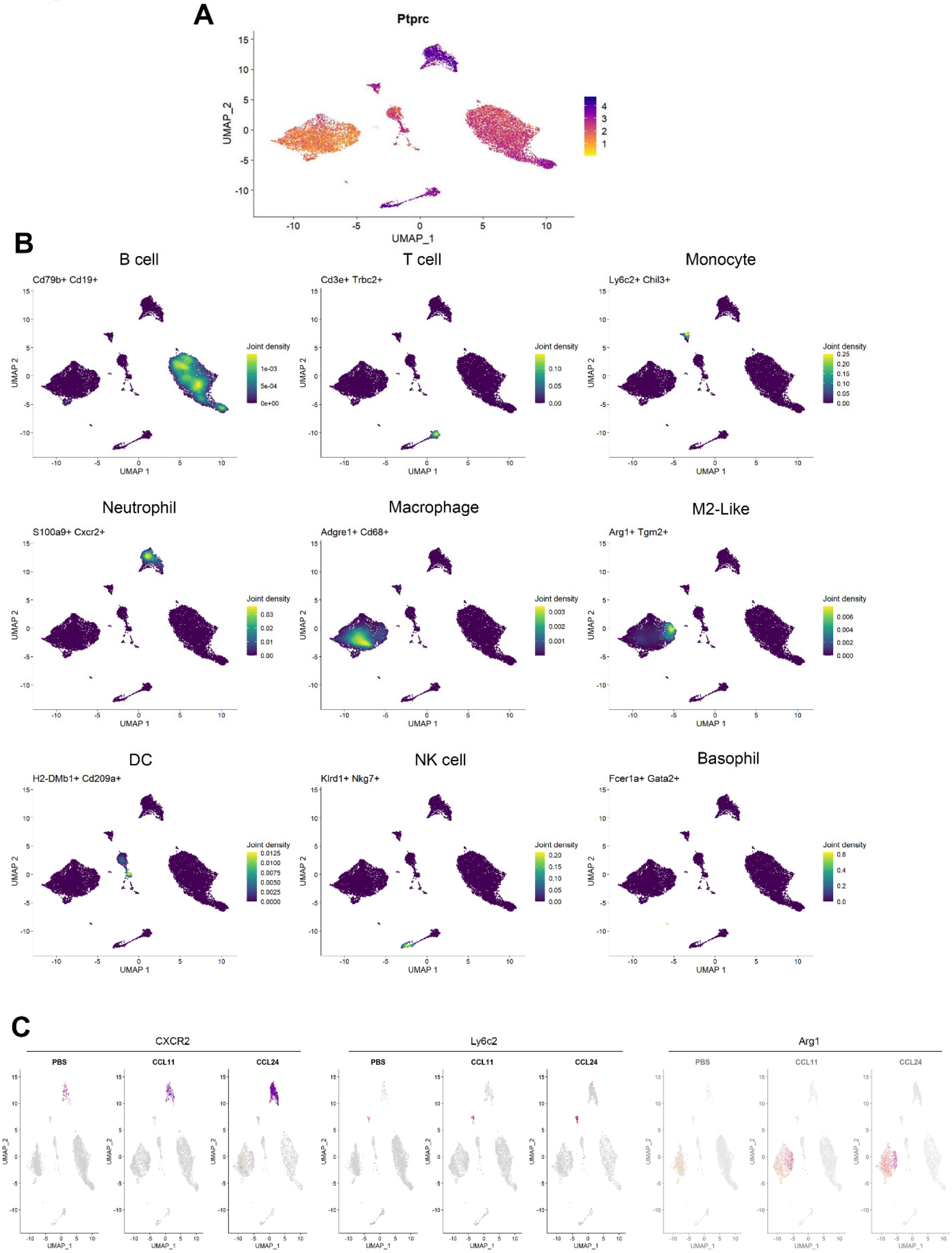
A
Ptprc
UMAP_2
UMAP_1
B
B cell
Cd79b+ Cd19+
T cell
Cd3e+ Trbc2+
Monocyte
Ly6c2+ Chil3+
Neutrophil
S100a9+ Cxcr2+
Macrophage
Adgre1+ Cd68+
M2-Like
Arg1+ Tgm2+
DC
H2-DMb1+ Cd209a+
NK cell
Klrd1+ Nkg7+
Basophil
Fcer1a+ Gata2+
Joint density
C
CXCR2
Ly6c2
Arg1
PBS
CCL11
CCL24

Fig S5. scRNA-seq of peritoneal fluid following injection of PBS, CCL11 or CCL24. (A) Expression of Ptprc (CD45) showing a ubiquitous expression pattern. (B) Joint density plots of canonical cell-type marker pairs. (C) Feature plots of selected genes, demonstrating the difference in expression by treatment.

Fig S6

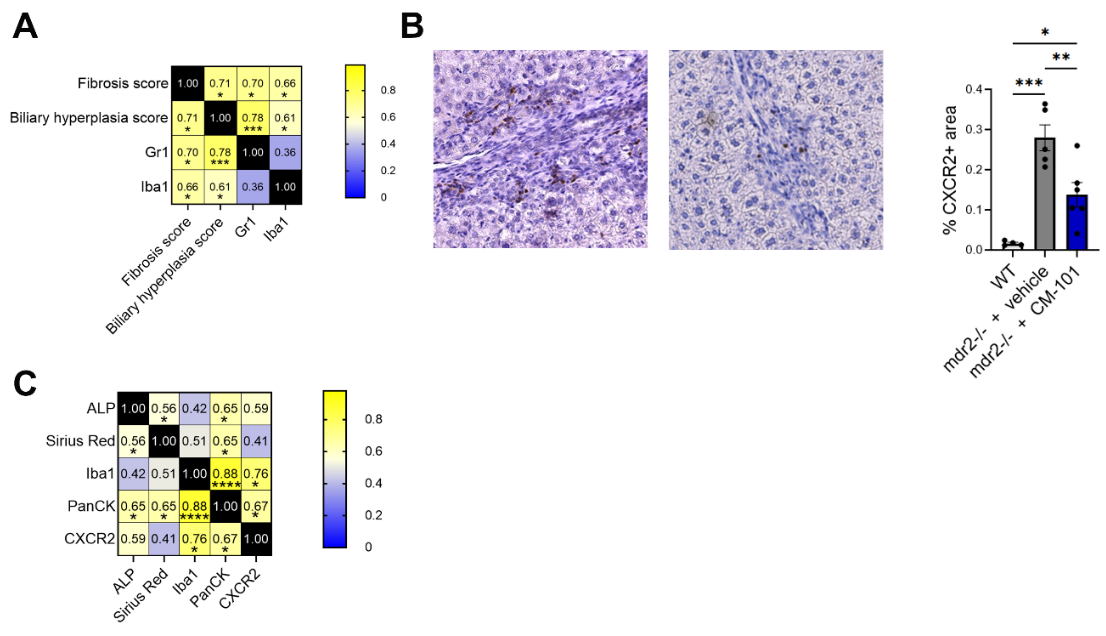

Fig S6. CCL24 neutralization with CM-101 attenuated experimental sclerosing cholangitis progression. (A) Correlation (Spearman) matrix in ANIT-cholestasis model between fibrosis score, biliary hyperplasia score, Iba1 positive stain area and Gr1 positive stain area. (B) CXCR2 IHC in peribiliary area of Mdr2$^{-/-}$ cholestasis model. Data are represented as mean ± SEM (n = 4-6). (C) Correlation (Spearman) matrix in of Mdr2$^{-/-}$ cholestasis model between serum ALP levels, Sirius Red positive stain area, Iba1 positive stain area, PanCK positive stain area and CXCR2 positive stain area. *, $p < 0.05$; **, $p < 0.01$; ***, $p < 0.001$; ****, $p < 0.0001$.

Fig S7

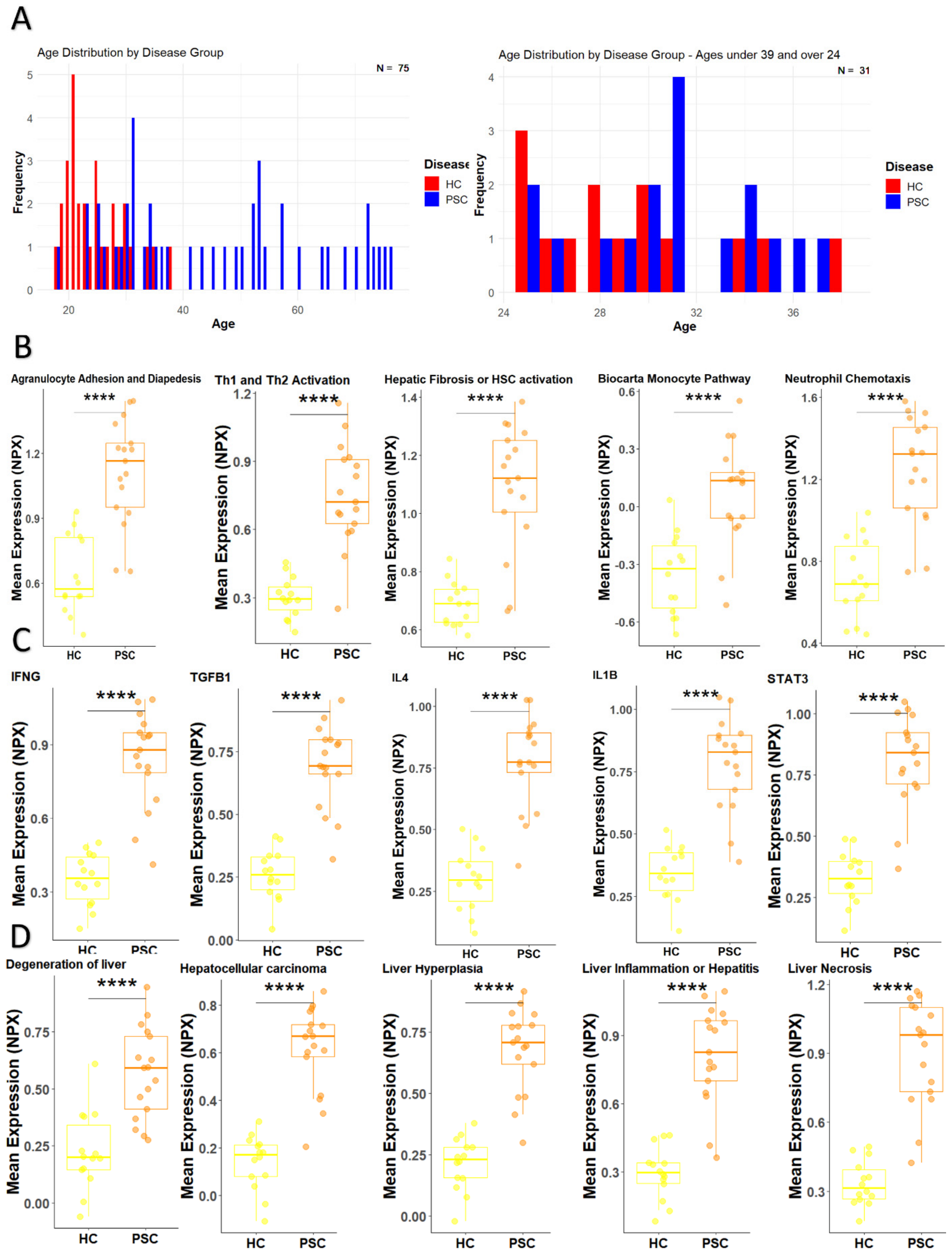

A
Age Distribution by Disease Group
N = 75
Frequency
Age
Disease
HC
PSC
Age Distribution by Disease Group - Ages under 39 and over 24
N = 31
B
Agranulocyte Adhesion and Diapedesis
Th1 and Th2 Activation
Hepatic Fibrosis or HSC activation
Biocarta Monocyte Pathway
Neutrophil Chemotaxis
Mean Expression (NPX)
****
HC
PSC
C
IFNG
TGFB1
IL4
IL1B
STAT3
D
Degeneration of liver
Hepatocellular carcinoma
Liver Hyperplasia
Liver Inflammation or Hepatitis
Liver Necrosis

Fig S7. PSC-related canonical pathways, upstream regulators and liver toxicity functions are upregulated in a subset of age-matched population. (A) Histogram showing the age distribution of healthy controls (red) and PSC patients (blue) of the entire study population (on the left) and the age-matched subset (on the right). (B) Boxplots of the mean expression (in NPX) of IPA canonical pathways, limited to the age matched subset population. (C) Boxplots of the mean expression (in NPX) of IPA upstream regulators, limited to the age matched subset population. (D) Boxplots of the mean expression (in NPX) of IPA toxicity functions, limited to the age matched subset population. Boxes represent interquartile ranges with medians (n = 14-17). ****, $p<0.0001$

	r Pearson
CCL24	1
TIMP3	0.6837
DPT	0.524211
APOH	0.51403
MB	0.50145
CCL7	0.499525
CXCL9	0.496228
CXCL10	0.483641
HSPB6	0.468124
IL12B	0.467865
CDCP1	0.458641
PTPRC	0.448785
GZMA	0.442174
LILRA2	0.440227
SORT1	0.438523
PAMR1	0.438169
VEGFA	0.438037
SELE	0.437231
NID2	0.432083
TNFRSF9	0.429871
GLIPR1	0.420759
CCL11	0.418842
PRG2	0.418702
LDLR	0.416172
COL24A1	0.414657
ITIH4	0.409991
SLC39A5	0.406972
PROS1	0.40494
IL12A_IL12B	0.403578
STAB2	0.403545
TREM2	0.403067
BGLAP	0.402413
RNASE4	0.399916
KCNC4	0.396465
MARCO	0.39161
CCL16	0.390684
PRG3	0.390535
NHLRC3	0.388905
LGALS1	0.388321
NDUFS6	0.386605
CRTAC1	0.380419
SSC5D	0.380055
ADGRF5	0.378654
TOMM20	0.37852
CD80	0.378502
CFI	0.376624
KLRF1	0.37586
TGFBR1	0.375717
PRCP	0.374769
ITGB2	0.373823

Supplemental Table S1: Top 50 circulating proteins that correlate with CCL24

	Healthy controls	PSC (UCL Bio-Bank and SPRING trial)
**N**	14	17
**Age [y], median (range)**	28.5 (25-38)	31 (25-37)
**Duration since diagnosis [y], median (range)**	NA	2.1 (0-10.1)
**Male, n (%)**	14 (100)	12 (71)
**IBD any, n (%)**	0 (0)	10 (59)
**ALP [U/L], median (range)**	66.5 (46-95)	297 (63-1064)
**ALT [U/L], median (range)**	16.5 (8-36)	127 (31-256)
**AST [U/L], median (range)**	19 (15-34)	72 (20-218)
**Bilirubin [mg/dL], median (range)**	11 (7-18)	15 (5-38)
**Fibroscan, median (range)**	NA	8.8 (5.0-13.6)
**ELF, n, median (range)**	NA	12, 9.03 (7.85-11.31)

Supplemental Table S8: Characteristics table of age-matched groups
